# Tracking and monitoring the health workforce: a new human resources information system (HRIS) in Uganda

**DOI:** 10.1186/1478-4491-9-6

**Published:** 2011-02-17

**Authors:** Julie C Spero, Pamela A McQuide, Rita Matte

**Affiliations:** 1 IntraHealth International, Chapel Hill, North Carolina, USA; 2 IntraHealth International, Kampala, Uganda

## Abstract

**Background:**

Health workforce planning is important in ensuring that the recruitment, training and deployment of health workers are conducted in the most efficient way possible. However, in many developing countries, human resources for health data are limited, inconsistent, out-dated, or unavailable. Consequently, policy-makers are unable to use reliable data to make informed decisions about the health workforce. Computerized human resources information systems (HRIS) enable countries to collect, maintain, and analyze health workforce data.

**Methods:**

The purpose of this article is twofold. First, we describe Uganda's transition from a paper filing system to an electronic HRIS capable of providing information about country-specific health workforce questions. We examine the ongoing five-step HRIS strengthening process used to implement an HRIS that tracks health worker data at the Uganda Nurses and Midwives Council (UNMC). Secondly, we describe how HRIS data can be used to address workforce planning questions via an initial analysis of the UNMC training, licensure and registration records from 1970 through May 2009.

**Results:**

The data indicate that, for the 25 482 nurses and midwives who entered training before 2006, 72% graduated, 66% obtained a council registration, and 28% obtained a license to practice. Of the 17 405 nurses and midwives who obtained a council registration as of May 2009, 96% are of Ugandan nationality and just 3% received their training outside of the country. Thirteen per cent obtained a registration for more than one type of training. Most (34%) trainings with a council registration are for the enrolled nurse training, followed by enrolled midwife (25%), registered (more advanced) nurse (21%), registered midwife (11%), and more specialized trainings (9%).

**Conclusion:**

The UNMC database is valuable in monitoring and reviewing information about nurses and midwives. However, information obtained from this system is also important in improving strategic planning for the greater health care system in Uganda. We hope that the use of a real-world example of HRIS strengthening provides guidance for the implementation of similar projects in other countries or contexts.

## Background

In all countries, health systems rely on their health workforce in order to deliver effective, efficient, and high quality health services. Without strong human resources for health (HRH), health systems are unable to provide primary health and preventive services, diagnose and treat patients, and administer life-saving pharmaceuticals. Nurses, the first line of health care in most health systems, are in critically short supply throughout the globe. This shortage is of grave concern, as nursing skills and labour are crucial in order to achieve the Millennium Development Goals and to provide fundamental health services [1].

Uganda is one of several sub-Saharan African countries that have experienced a shortage of health workers [2]. Consequently, hospitals and health facilities have experienced a shortage of qualified staff [3]. In 2009, the nursing vacancy rate was as high as 53% in public hospitals and the number of available staff was far below the nationally recommended norm [4].

Managers and health planners need information about the size, composition, skill sets, training needs, and performance of the public health workforce in order to make informed, well-timed decisions [5,6]. The absence of this information can have negative consequences on health system functioning. In fact, the lack of accessible and reliable health workforce information has been cited as one of the key factors responsible for the shortage of nurses in sub-Saharan Africa [7]. In recognition of the importance of reliable data, the development and use of HR information and management systems has been recommended as an attainable and cost-effective strategy to address workforce shortages and improve public health in developing countries [6-8]. At the 2008 East, Southern and Central Africa Health Community (ECSA HC) Forum on Best Practices, recommendations were made and subsequently, a resolution was passed by the ECSA Health Ministers to support the development of comprehensive human resources information systems (HRIS) at training institutions, regulatory bodies and employers, and to build capacity for HRIS use to inform policy and decision-making [9].

In 2004, despite the existence of a variety of independent sources of health workforce data, (including censuses and other national surveys, the Ministry of Health (MOH), district level sources, independent research studies, and health professional council data) [10], Uganda was described as being in need of better information about the state of its health workforce [11]. Although a health management information system (HMIS) had previously been implemented with somewhat limited success due to technological and organizational challenges, an information system specific to the health workforce was lacking [12,13].

Uganda has rich sources of data in each of its four health professional councils, including the Uganda Nurses and Midwives Council (UNMC). The UNMC is an official body charged with regulating standards for nursing and midwifery in Uganda. The UNMC is an arm of the MOH that makes recommendations to the Government of Uganda regarding issues pertinent to nurses and midwives [14]. The Council's authority and scope is based on the 1996 Uganda Nurses and Midwives Act. The Council has several functions, including setting continuing professional education requirements, providing and tracking nursing and midwifery registrations and licenses to practice, and serving in a disciplinary role in cases of professional misconduct. The UNMC used to be responsible for accrediting schools of nursing, but a later statute has since granted the Ministry of Education and Sports authority to govern nursing and midwifery training curricula, examinations, and training institution accreditation. Legal structures within the country have determined that the most current law takes precedence until both statutes are harmonized. The UNMC also provides recommendations and contributions to the Ministry of Education regarding nursing and midwifery training and accredited curricula.

One of the UNMC's tasks is to track training information about nurses and midwives throughout Uganda, from pre-service training through licensure. Following graduation from a particular training program, all nurses and midwives from the public, private, faith-based (FBO), and nongovernmental (NGO) sectors are mandated to register with the Council. Uganda law states that nurses and midwives must have a license in order to practice nursing or midwifery, which must be renewed every three years following completion of the requisite number of continuing professional education credits. Prior to decentralization, the licensure requirement was not routinely exercised by employers because newly qualified nurses and midwives would receive an automatic posting immediately following examination results and would register at the UNMC at their leisure. However, employers are now demanding verification of Council registration prior to hiring. Thus, the UNMC serves as a repository of information, including licensure and registration data, which can be verified prior to employment. (However, it is critical to note that the Council has not yet had adequate staff to efficiently and effectively fulfil the function of ensuring that all nurses and midwives are licensed and registered at time of hire.)

Prior to 2005, the UNMC maintained all of their workforce data using a system of paper files. However, the paper-based system was infrequently updated, records were subject to being misplaced or lost, and locating information about individuals was time-consuming. Most importantly from a health planning perspective, the paper filing system did not provide a way to aggregate and analyze the data. Therefore, the Council had no way of accurately determining how many nurses and midwives had been registered, much less where they were deployed. The Senior Nursing Officer at the UNMC stated, "I used to feel guilty when requested to talk about the total number of qualified nurses and midwives in the country because I knew that we did not have accurate data" [15]. Simply put, HR information in Uganda was not readily available or accessible to those who needed it for health planning and management decisions.

The UNMC desired an electronic database with the ability to quickly update, aggregate, and analyze HRH information. To achieve this objective, the Uganda MOH and UNMC partnered with the Capacity Project, a USAID-funded global initiative led by IntraHealth International to strengthen the health workforce in developing countries. The goal of this collaboration was to transition the UNMC's records from the paper files into an electronic HRIS capable of aggregating the data and creating reports. Although the Project conducted HRIS strengthening activities in all four of Uganda's professional councils (including the Uganda Medical and Dental Practitioners Council, the Allied Health Professional Council, and the Uganda Pharmacy Council) and the MOH, the focus of this paper is the HRIS strengthening process applied at the UNMC.

### The HRIS strengthening process

#### Building HRIS stakeholder leadership

Prior experience in health information system strengthening has demonstrated the need for advocacy and continuous dialogue between decision-makers and information system implementers in order for system strengthening to be successful [16]. In Uganda, the Project sought to create an environment where stakeholders from a variety of perspectives could collaborate and share ideas about HRIS implementation. The Project's first step in HRIS strengthening was to bring together all leaders and decision-makers that would have an interest in the HRIS, via a stakeholder leadership group (SLG). The purpose of the Uganda SLG was to determine the specific priorities the system needed to address and to become the driving force motivating HRIS implementation. The SLG, known as the Health Workforce Advisory Board (HWAB), included representatives from the MOH, the four national health professional councils, training institutions, NGOs, and Project staff. HWAB members met and communicated regularly to address implementation challenges, identify necessary customizations and reports, and make decisions as needs arose.

It should be noted that when the Capacity Project started work in Uganda, a separate, Government-recognized Human Resources Technical Working Group (HRTWG) already had an official charter. The purpose of the HRTWG was to meet formally to discuss HR issues and provide input to the MOH and other ministries; however, although the HRTWG existed on paper, the group had not met in several years. Throughout the time period when the HWAB was established and held regular meetings, the Capacity Project simultaneously supported the revitalization of the official HRTWG. Eventually, the HRTWG began to hold formal meetings. Rather than having two separate groups, the HWAB subsequently became a recognized subcommittee of the HRTWG. As of the time of this writing, consultations are on-going for the purpose of developing the HWAB into an HRH Observatory. The HWAB holds regular quarterly meetings and meets more frequently when required. The HWAB continues to make recommendations to the HRTWG regarding HRIS implementation.

#### Strengthening ICT infrastructure

The next step in HRIS strengthening was to assess and improve the UNMC's information and communication technology (ICT) infrastructure. This process included an evaluation of the existing ICT hardware, software, and web connectivity, all of which were upgraded in order to be able to operate and sustain the new HRIS. A Local Area Network (LAN) was installed at the UNMC and staff received training about the administration and maintenance of the upgraded ICT system.

#### Developing an HRIS software solution

Following ICT upgrades, iHRIS Qualify was installed at the UNMC. iHRIS Qualify is an Open Source software program, designed for use at a health professional regulation authority, which can be used to track information about health workers from pre-service training through registration and licensure. (The term 'Open Source' refers to software applications that are distributed under an Open Source license, meaning that anyone can use, copy, share, or modify the software without paying a licensing fee.) The software is web-accessible, server-based, regularly backed up, and can be accessed by multiple users at once. Data are stored in a central database.

#### Promoting a culture of evidence-based decision making

While a new HRIS provides substantial benefit, the system itself has little meaning out of context [16-18]. For this reason, HWAB members, UNMC staff, and other stakeholders took part in an interactive workshop in June 2007 that enabled participants to practice decision making, analysis, and communication skills. Outcomes of the workshop included a deepened understanding of HRIS strengthening, experience and training with the HRIS software, development of practical skills on HRH needs and HRIS implementation, creation of action plans for continued HRIS strengthening, and development of a strategy for HRIS sustainability.

#### Building HRIS capacity

To ensure system sustainability, UNMC staff, including system administrators, data entry clerks, managers, analysts, and decision-makers received training on the development, maintenance, and continued use of the HRIS software, as well as general training on data quality and project management. The goal of these one-on-one and group training initiatives was to ensure that UNMC staff would be equipped to fully support, use, and continue to improve the HRIS once project support ended.

#### Ensuring data quality and security

The need for good data quality was emphasized during trainings with HRIS staff and data collectors. Data collection and entry processes were put in place with the goal of improving and maintaining data integrity. To ensure data quality, data were entered using pull-down menus rather than having data entry specialists manually type information into collection forms. The UNMC also adopted a new data validation process following system implementation. When a nurse physically comes to the Council for registration, licensure, or other purposes, he or she reviews either a printout or an on-screen copy of his or her personnel information and can verify the data. At the time of this writing, the UNMC's Commissioner of Nursing is working with district health offices to validate information in the HRIS. The Commissioner of Nursing prints hard copies of district staff lists, and uses this information to determine whether the nurses and midwives working in the district have registered with the UNMC and have renewed their licenses.

Several security measures were implemented to maintain data confidentiality. Password protected logins were assigned so that only authorized users would be able to access the system. Roles were assigned to users in order to control who had the ability to enter, update, and generate reports. For auditing purposes, the system logged the username, date, and time any data were entered or changed. Finally, the UNMC has also been developing a data use agreement, to be used when sharing the HRIS data with agencies external to the Council. This policy, created with key stakeholders and local legal authorities, will be used to protect data confidentiality and to assure the various stakeholders that the data will not be used inappropriately.

The goal of the remainder of this paper is to demonstrate how HRIS data can be used to address health workforce planning questions via an initial analysis of the UNMC training, licensure, and registration records.

## Methods

Our analysis relied on secondary data provided by UNMC training, licensure, and registration records. The Council maintained historical records of registration dates, exam results, and other pertinent information for nurses and midwives who had physically come to the UNMC offices to register. In addition, principal tutors submitted the names of all new students to the Council and these students obtained an index number within a month (note: this practice is no longer in effect since the Ministry of Education has taken over training nurses). UNMC data entry clerks transferred paper records dating from 1970 onward into iHRIS Qualify. Initial data entry was completed in March 2009. At the time of writing this manuscript, data entry of present day training, licensure, and registration data is ongoing.

The present descriptive study included records for nurses and midwives who entered training or registered with the Council between 1970 and 23 May 2009. (Please see Additional File 1 for a list of data fields collected in the UNMC HRIS.) Data were analyzed using the SPSS version 16.0 statistical analysis software [19]. In order to avoid confusion on the part of the reader, we wish to clarify the distinction between a UNMC registration and the 'registered' cadre of nurses and midwives. Following the completion of any nursing or midwifery training program, all nurses and midwives are required to obtain a one-time registration for that training at the UNMC. However, when speaking about cadre classifications, the terms 'enrolled' and 'registered' refer not to UNMC registration, but rather less advanced and more advanced levels of training, respectively. For example, a 'registered midwife' would have completed a more advanced training than an 'enrolled midwife,' but both midwives would be required to obtain a UNMC registration following completion of training and passing the examination. The term 'licensed' means that a nurse or midwife has obtained a license from the UNMC that allows her to practice nursing or midwifery. Licenses must be renewed every three years and a mandatory continuing professional education requirement must be completed prior to renewal.

Following the completion of data entry into the HRIS and prior to data analysis, three searches for duplicate records were conducted. Duplicates were identified based on matching surnames, first names, other names, and dates of birth. UNMC staff verified potential duplicates in the electronic database against the hard copy records. For cases in which paper records were reviewed and it was not possible to determine whether the records represented two separate individuals with the same name or duplicate hard copy records for a single individual, both records were retained in the database.

To avoid double counting, we also removed known duplicates from the database. All records (N = 23) previously marked "duplicate" or "deleted" by data entry clerks were removed. In addition, all records (N = 245) without a training record ID were removed from the database, as none of these individuals entered nursing or midwifery training or obtained index numbers. We believe these records were entered into the system erroneously by data entry clerks, who did not have the necessary access levels to delete any records from the system.

To further ascertain data quality, frequencies were run on all data fields to identify and eliminate obvious outliers due to errors in data entry. Analyses were also conducted on all dates to ensure that dates were entered in a way that made sense chronologically (e.g. ensuring that dates of birth preceded dates of training and ensuring that dates of training at lower levels preceded dates of training at more advanced levels). In cases where data appeared to be in error, comparisons were made to hard copy records. However, it should be noted that in many cases, the hard copy records themselves were incomplete or were filled out incorrectly. The hope of the UNMC is that once the UNMC's new on-site verification process (during which individual nurses and midwives review a print-out of their record from the UNMC database and recommend updates if needed) becomes routine, the number of errors in the database will decrease over time.

Variables of interest in this study included demographic data, such as gender and nationality. In addition, the study examined data related to entering training, graduating, registering with the Council, and cadre classification. Basic descriptive statistics were used to examine the characteristics of the nurses and midwives in the dataset. To our knowledge, this study presents the first analysis of the most comprehensive data available on the nursing and midwifery workforce in Uganda.

## Results

**Figure 1:**
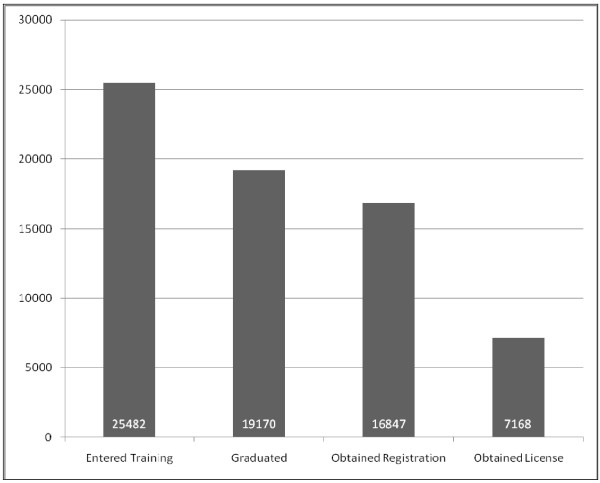
Number of nurses and midwives who entered training in Uganda before 2006, graduated, obtained a council registration, and became licensed (N = 25 482)

The data indicate that, as of 23 May 2009, a total of 26 046 people in Uganda have entered nursing or midwifery training (this number includes 527 nurses and midwives who received training outside of Uganda). Training programs typically last 3 years from intake to graduation. To determine completion rates for training programs, we first limited the dataset to nurses and midwives who entered training before 2005 (N = 25 482). The nurses and midwives who did not report a training intake date (N = 533) were not included in this dataset, nor were the nurses and midwives who reported a training intake date in 2006 or later (N = 31). Of those who reported a training intake date prior to 2006, 19 170 graduated and 16 847 obtained a council registration. Licensure data, available beginning in 2005, indicates that approximately 43% of the registered nurses and midwives (N = 7168) obtained a license to practice from the UNMC. Please see Figure 1 for more detail.

Other concerns regarding the deployment of nurses and midwives include both training completion rates and UNMC registration rates for those who begin nursing or midwifery training. Nursing education involves the investment of limited resources, including funding, instructor time, training materials, etc. To ensure that resources are used as efficiently as possible, data from the UNMC database can be used to target specific areas of need or locations in which training completion rates are lower than desired. For example, graduation rates from nursing and midwifery training programs can be disaggregated by training institution. This information is reported in Table 1. Note that this table only includes data from a nurse's or midwife's initial training and does not include information related to additional trainings begun after completion of the first training. For instance, 100% of the students who attended Mulago Health Tutors College (N = 40), Makerere University (N = 57), or Mbarara University (N = 21) graduated from the training program. Only 60% (N = 20) of the students at the Mbarara School of Enrolled Midwifery graduated, but 100% of them obtained a UNMC registration. Nsambya had the largest number of students who entered training (N = 3014), the majority of whom graduated (79%, N = 2383) and obtained a UNMC registration (68%, N = 2059). On the other hand, some schools with a smaller student body had a much lower rate of graduation. For example, 175 students entered training at the Jinja International School of Health Sciences, but only 7% of the nurses and midwives (N = 12) reported a date of graduation.

**Table 1:** Graduation and registration rates by training institution for nurses and midwives who entered training between 1970 and 2005 (N = 25 482) (Data included for earliest training only)

Name of training institution	Number entered training	Per cent graduated	Percent with UNMC registration
Kampala	1	100.00%	100.00%
Mulago Health Tutors College	40	100.00%	100.00%
Makerere University	57	100.00%	96.49%
Mbarara University	21	100.00%	95.24%
Rubaga	586	86.18%	74.23%
Mutolere School of Nursing & Midwifery	309	85.44%	79.94%
Arua School of Enrolled Comprehensive Nursing	1,346	84.70%	78.53%
Lacor School of Nursing	862	80.16%	71.35%
Kabale School of Enrolled Comprehensive Nursing	1,781	79.56%	62.94%
Mulago School of Nursing	2,442	79.32%	64.91%
Mengo	1,883	79.18%	69.14%
Nsambya	3,014	79.06%	68.31%
Nyakibale School of Nursing & Midwifery	747	77.91%	67.34%
Masaka School of Comprehensive Nursing	898	76.06%	57.46%
Ibanda School of Midwifery	338	76.04%	75.74%
Matany School of Nursing	481	74.64%	70.48%
Ngora School of Nursing & Midwifery	715	74.55%	67.83%
Villamaria School of Nursing	654	73.70%	69.11%
Virika School of Enrolled Comprehensive Nursing	993	73.31%	67.17%
Kalongo School of Midwifery	687	72.93%	61.57%
Kamuli School of Midwifery	771	72.89%	67.57%
Soroti School of Comprehensive Nursing	595	72.77%	63.36%
Butabika School	890	72.02%	69.21%
Jinja School of Nursing	1,755	71.74%	64.62%
Lira School of Enrolled Comprehensive Nursing	1,719	70.97%	62.19%
Kuluva School of Nursing	362	66.57%	65.75%
Gulu School of Midwives	73	65.75%	54.79%
Kagando School of Nursing & Midwifery	224	63.84%	62.95%
Mbarara School of Enrolled Midwifery	20	60.00%	100.00%
Kampala International University	49	59.18%	40.82%
Kisiizi School of Nursing	179	52.51%	55.31%
Kiwoko School	193	50.26%	50.78%
Ishaka Adventist School of Nursing	180	40.56%	40.56%
Aga-Khan University	29	37.93%	34.48%
Rakai School of Community Health Nursing	75	16.00%	14.67%
Jinja International School of Health Sciences	175	6.86%	7.43%
Nakaseke Mwagalwa	25	0.00%	0.00%
Not Reported	313	67.41%	60.38%

Total	25 482	75.23%	66.11%

The remainder of this analysis is concerned with the 17 405 nurses and midwives who obtained a UNMC registration. Along with the 16 847 nurses and midwives who entered training prior to 2006 and obtained a Council registration, there are 558 additional nurses and midwives included here that were not included in the prior analyses. This addition takes into account the 527 nurses and midwives who completed training outside of Uganda (and therefore did not report a training intake date) as well as the 31 nurses and midwives who reported entering training in Uganda after 2006. These are the health workers who most likely compose the actual nursing and midwifery workforce in 2009, as only those nurses and midwives with a Council registration are legally eligible to work. However, we recognize that not all nurses and midwives with a Council registration are active in the workforce; therefore, these results should be interpreted as approximations rather than definitive numbers.

The vast majority of the nurses and midwives (96.05%, N = 16 717) were Ugandan nationals. Just 3.55% reported a nationality other than Uganda, most frequently Kenya (N = 186), Germany (N = 71), the United States of America (N = 39), and the United Kingdom of Great Britain and Northern Ireland (N = 38). Seventy of the nurses and midwives did not report a nationality. As noted above, just 527 nurses and midwives in the dataset were trained in countries other than Uganda. Not surprisingly, the most frequently reported outside countries of nationality were similar to the most frequently reported outside countries of training. Kenya was the most frequently mentioned outside country of training (N = 97), followed by the United Kingdom (N = 93), Germany (N = 72), the United States of America (N = 47), and the United Republic of Tanzania (N = 32). Note that some nurses and midwives obtained a UNMC registration for multiple trainings outside of Uganda. None of the nurses and midwives who were trained outside of Uganda reported a date of entering training, graduating, or taking the qualifying exam. However, all 527 nurses and midwives who received training outside of Uganda did report a date of registration with the UNMC and 66 reported dates of licensure.

Approximately 13% (N = 2291) of the nurses and midwives obtained Council registrations for two or more trainings. For example, a worker may have obtained a Council registration following an initial training as an enrolled midwife, then again after completing a second training at the registered nurse level. Few workers (N = 205) obtained a Council registration for 3 trainings, and very few (N = 13) obtained a registration for four trainings. No worker in the database obtained more than four different registrations. In total, there are 20 141 different Council registrations in the database, including 12 807 registrations for nursing trainings and 7195 midwifery trainings. Note that the total number of nurses and midwives in this dataset remains 17 405; however, there are 20 141 registrations because 2291 of these nurses and midwives obtained more than one registration at the UNMC following completion of one or more trainings. Please see Table 2 for a complete list of trainings by cadre.

**Table 2:** Trainings with a UNMC registration

Cadre	Number of trainings
Enrolled Nurse	6916
Enrolled Midwife	4945
Registered Nurse	4310
Registered Midwife	2250
Registered Comprehensive Nurse	389
Registered Mental Health Nurse	366
Enrolled Mental Health Nurse	299
Enrolled Comprehensive Nurse	240
Registered Health Tutor	139
Registered Paediatric Nurse	126
Registered Public Health Nurse	117
Registered BScN	44
**Total**	20 141*

*not unique individuals

The largest number of trainings with a Council registration are at the enrolled nurse level (N = 6916, 34.34%), followed by enrolled midwife (N = 4945, 24.55%), registered nurse (N = 4310, 21.40%), and registered midwife (N = 2250, 11.17%). The more specialized trainings make up only 8.54% (N = 1720) of the total number of registrations. There are fewer specialized trainings in the Ugandan nursing and midwifery workforce because most of the specialty training programs are relatively new. Moreover, many students first complete a basic training at either an enrolled or registered level before beginning training in a specialty.

Since training at the registered level is more advanced than training at the enrolled level, for the purposes of this paper, nurses who had obtained a council registration for trainings at both the 'enrolled' and 'registered' levels were grouped in the 'registered' level. When using this classification method, the majority of the nursing and midwifery workforce has been trained at the enrolled level (60.06%, N = 10 454); there are 6913 nurses and midwives at the registered level in the Ugandan health workforce.

Approximately 88% (N = 15 334) of nurses and midwives were female, 11.5% (N = 2007) were male and the remaining 64 did not report a gender. Chi-square statistics demonstrated that there were no significant differences in gender distributions between the enrolled and registered levels (χ^2 ^= 0.455, df = 1, n.s.).

### Limitations

Several limitations should be considered when interpreting the results of this study. As previously mentioned, prior to analysis we identified a list of potential duplicates based on surname, first name, other names, and date of birth and compared these with the hard copy records at the UNMC. However, we may have missed some additional duplicates. Duplicate records may have been created if a nurse's name was misspelled or legally changed following marriage. Such mistakes were nearly impossible to track when updating the paper-based system.

Assignment of a computer-generated unique identifier for each individual in the nursing and midwifery workforce occurred after the implementation of the electronic HRIS, since no unique identifier existed in the paper files. In the future, the license number will serve as the unique identifier which should help to reduce future duplicate entries in the system. (The license number remains the same over the course of a nurse or midwife's career, but the expiration date changes after the three year renewal.)

Second, data were entered into the HRIS from historical paper records, which may not have been updated when nurses retired, left the public sector, moved, or passed away. Therefore, the database may contain information from individuals who have exited the workforce and may overestimate the number of nurses and midwives available to serve the public.

Third, we included 82 nurses and midwives in the database who appeared to be over the age of 60, the retirement age in Uganda. We decided to retain the information collected for these nurses in the dataset, as some people in Uganda continue to work past the age of 60 in non-public sector jobs. However, we recognize that some of these nurses may have retired, which may not have been reflected in their personnel files. Therefore, we may have inadvertently included data for nurses who are no longer active in the workforce.

Fourth, we used Council registration rather than licensure to estimate the number of nurses and midwives in the Ugandan workforce. Again, it is likely that some of these 17 405 nurses and midwives have retired, died, migrated, or otherwise left the workforce. Licenses to practice, which must be renewed every 3 years, have only been recorded in the database if they were obtained in 2005 or later. We decided to analyze data for all nurses and midwives with a UNMC registration rather than a license, so as not to underestimate the size of the available workforce. We recognize that some nurses and midwives who have not obtained a registration with the UNMC may be active in the workforce, albeit illegally. Consequently, the numbers reported in this paper should be treated as approximations.

Finally, the large amount of missing data in the system limited our ability to infer information about some health worker characteristics. For example, in the dataset of nurses and midwives with a registration, 23.09% (N = 4018) did not report birth dates, 96.78% (N = 16 845) did not report marital status, 38.21% (N = 6651) did not report information about home district, and 28.96% (N = 5040) did not report information about birth district. Because some of the paper records entered into the system were incomplete or illegible, it was not possible to remedy these gaps in data. Additionally, since birth dates were missing in 23% of the cases, it was not possible to determine the number of registered nurses and midwives 60 years old or less.

The UNMC is clearly still in the beginning stages of a transition from an entirely paper-based system to an electronic HRIS. Because the data are largely limited to the historical records, unless a nurse or midwife has verified the information in person, it is not possible to use these data to definitively determine whether that individual is currently active in the workforce. This uncertainty is a major limitation of the dataset and should be considered when interpreting our results. However, at the same time, the HRIS represents an enormous step forward for the UNMC and the larger Ugandan health system. Previously, this workforce information was only accessible in hard copy files; now, these data are electronically available and can be aggregated and analyzed for decision-making. It is the hope of the HWAB and the UNMC that as the system continues to be used and nurses and midwives regularly review and update their information, the data in the system will become increasingly more reliable and accurate.

## Discussion

The UNMC's HRIS is a valuable source of information on Uganda's nursing and midwifery workforce. Health planners are now able to assess the skill mix of the national nursing and midwifery workforce as well as to examine its composition based on demographic variables, like gender. The data provide an estimate of total number of each type of training that Ugandan nurses and midwives have received. The majority of trainings with registrations have been at the enrolled level, the most basic and general level of nursing and midwifery instruction. However, the data also allow planners to estimate the number of registrations for more specialized trainings, such as the 665 trainings received for mental health nursing.

The data can also be used to determine district-level training needs and gaps. Prior research by Nguyen et al. (2008) indicated that Ugandan nurses born in rural areas were more likely to continue to work in those areas following completion of training [20]. Additionally, a study on health workforce retention in Uganda indicated that health workers tend to work in the region in which they were born or completed their training [21]. Once information on district of birth and district of residence are more complete, UNMC planners will be able to use the system to plan for workforce needs at the district level and to inform pre-service training recruitment strategies and policies.

Data on graduation and registration rates from training institutions can be used to identify successful training programs. Follow-up studies can then be conducted to determine the reasons why some programs graduate a greater percentage of students than do others. Lessons learned from the successful programs can be applied to institutions where graduation rates are not as high.

Our analyses demonstrated that the rates of licensure were very low, due to the fact that licenses to practice were only recorded at the UNMC from 2005 onward. Legally, nurses and midwives should have an active license in order to practice in Uganda [14]. The Uganda Nurses, Midwives, and Medical Assistants Ordinance, which requires nurses and midwives to register with the Council prior to practicing, dates back to 1958. Limited resources have been put in place to enforce this law although employers are beginning to routinely insist on registration verification. Registration and licensure is difficult for many practicing nurses and midwives, particularly those from rural areas, due to the need to be physically present at the Council offices in Kampala for registration and license renewal.

During a phone conversation on 17 September 2010, Margaret Chota, the Commissioner of Nursing at the UNMC, noted that part of the reason hiring agencies have not routinely insisted on licensure as a prerequisite for hiring, despite the existence of the law, was that the data were not previously accessible. The HRIS at the UNMC now serves as a source of aggregated information that can be used to assist the regulatory authorities to enforce the legal mandates. Mrs. Chota noted that the UNMC plans to ensure that all nurses and midwives working for the government of Uganda meet the licensure qualification. In addition, in recognition of the importance of ensuring that all health professionals meet the legal requirements for practice, the MOH has established District Supervisory Authorities (DSAs) who represent the health professional councils at the district offices. The DSAs will work in collaboration with District Health Officers and other authorities to ensure that health workers are registered and licensed, regardless of whether they are working in the public, private, faith-based, or NGO sectors. According to Mrs. Chota, the HRIS database will be available at all districts to monitor the regulatory status of all health workers in each district. Since the majority of health workers are hired at the district level and not by the central MOH, the HRIS will be a useful tool in this decentralized system.

The ability to link records by a single license number, which can be used to identify individuals and link multiple trainings to a single identifier, will be a critical factor in ensuring that the HRIS remains up-to-date and useful. Having a single identifier ensures that nurses and midwives are not double counted if they attend multiple trainings, which is the case for almost a quarter of them. The HRIS will be the authoritative data source to track nurses across the public, private, NGO, and FBO sectors. Reports generated by the system should be triangulated the with other data sources such as the census.

The HWAB remained involved in the strategic direction and guidance of the UNMC's HRIS from its inception. The Commissioner of Nursing Officer, an HWAB member, has directly benefited from the system as it has enabled reports to be quickly generated from the UNMC's data. In addition, as mentioned previously, an electronic HRIS with aggregated licensure and registration data permits the Commissioner of Nursing Officer and the District Supervisory Authorities to verify applicant credentials at time of hire. During recruitment, in addition to using an MS Excel spreadsheet (instead of a manual process) to shortlist applicants for interviews, health worker registration numbers are verified against the data in the HRIS. Enforcing licensure will better enable the UNMC to verify that nurses and midwives working in Uganda have attained a minimum standard of training, knowledge, and skills prior to practicing, thereby promoting quality of care and preventing those with falsified records to practice.

It should be noted that the UNMC's HRIS is just one component of the larger system. The UNMC's data, along with the HRIS data from the Uganda Medical and Dental Practitioners Council, the Allied Health Professional Council, and the Uganda Pharmacy Council were used in an HRH Action Framework (HAF) evaluation to project the costs and resources required to staff up Uganda's health workforce to meet the national norms. These data were also used in an official evidenced-based supplement to the Uganda Human Resources for Health Strategic Plan 2005-2020, one of the components of the President's Master Plan for Accelerating Performance in the Health Sector [22-24].

On a broader level, the HWAB became an important forum for stakeholders to express their views and work collaboratively to further the progress of HRIS development among all councils. One of the outcomes of the HWAB was the creation of a semi-annual report that used the HRIS data to determine the number of filled and vacant positions in public hospitals and health centers throughout the country. Hard copies of the semi-annual report have been printed and used by the Commissioner of Nursing Office during supportive supervision visits to District Health Offices, in order to verify the registration and licensure status of nurses and midwives in those districts. The semi-annual report was used during meetings with the MOH, the Ministry of Public Service, and the Ministry of Finance, as an evidence-based advocacy tool to encourage increasing financial support for training greater numbers of nurses and midwives. The MOH has also used the semi-annual report to expedite recruitment. The report contains information about the number of health workers projected to retire. The MOH has used this information to post advertisements for positions before the retirees leave, which has reduced the gap time to hire replacement workers.

In addition, the HWAB developed an advisory relationship with the Human Resources Technical Working Group (HRTWG), a formal working group created by the Government to discuss national HR policies. The HRTWG advises the Government directly on policy and budgetary decisions regarding HRH issues throughout the country.

## Conclusions

The electronic HRIS added significant value to the UNMC's way of 'doing business'. Electronic records are easier to find and update, enabling Council staff to more efficiently verify a prospective employee's training qualifications. Checking a nurse's registration prevents unregistered nurses (who may not have graduated from school) and those with fraudulent credentials from obtaining employment. In addition, the system provides a way to ensure that nurses and midwives have completed the continuous professional development courses required to maintain licensure. This verification process enables the UNMC to fulfil its social contract of maintaining a minimum standard of nursing care, thereby instilling public confidence in the health care system.

At the time of this writing, the data from the UNMC database are being used to verify qualifications at the time of hire, to develop a semi-annual HR report, to advocate for training an increased number of health workers, and to expedite the recruitment process in the public sector. The system currently has substantial gaps in data accuracy and completeness. However, the existence of an electronic system with the ability to aggregate health workforce data for reporting and analysis represents a huge step forward from the former paper filing system. Furthermore, as new information is entered into the system, the database becomes increasingly refined, accurate, and complete. Information gleaned from the UNMC HRIS can be fed back into the information systems at the central MOH for planning and administration purposes beyond the nursing and midwifery workforce. Rather than a standalone program, the UNMC system is an important component of a larger, national HRIS. Once data on licensure are complete, the system can be used to determine whether the majority of nurses with a license are active in the workforce and whether they are eligible to apply for out-migration. Nevertheless, the database is not in itself a complete solution. To remain sustainable, an HRIS must be continuously updated and maintained. As of the time of this writing, the HRIS at the UNMC is under-utilized for routine operations. Future work should focus on designing new approaches to engage staff and stakeholders in fully utilizing the system. Building support for a culture that values evidence-based decision making is crucial to generate enthusiasm and forward momentum for such a system.

## Competing interests

The authors declare that they have no competing interests.

## Authors' contributions

JCS contributed to the study conception and design, analysis and interpretation of data, and drafting the manuscript. PAM contributed to the study conception and design, interpretation of data, and critical revision of the manuscript. RM contributed to acquisition of the data and the critical revision of the manuscript. All authors have read and approved the final manuscript.

## Supplementary Material

Additional file 1 List of Data Fields Collected in the UNMC HRIS Click here for file
