# Supplementary material for: Tracking and monitoring the health workforce: a new human resources information system (HRIS) in Uganda
Source: Hum Resour Health. 2011 Feb 17;9:6. doi: 10.1186/1478-4491-9-6 (PMC3049180; doi:10.1186/1478-4491-9-6)
Supplement: Additional file 1 — List of Data Fields Collected in the UNMC HRIS [file 1478-4491-9-6-S1.DOC]

## Additional file 1 – List of Data Fields Collected in the UNMC HRIS

Surname

First Name

Other Names

Nationality

Tribal Affiliation

Gender

Marital Status

Birth Country

Birth District

Birth Date

Home Country

Home District

Home County

Date of Record Verification

Outcome of Record Verification

Education Secondary School

Education Academic Level

Education Certificate Type

Training Index Number

Training Intake Date

Training Cadre

Training Graduation Status

Country of Training

Training Disruption Category

Training Disruption Reason

Training Disruption Date

Training Resumption Date

Exam Application Date

Exam Date

Exam Try (1^st^ try, retry, etc.)

Exam Results

Exam Number

Registration Number

Registration Application Date

Registration Date

Registration Practice Type

License Number

License Start Date

License End Date

License Suspended

Deployment Health Facility

Deployment Date

Out Migration Country

Out Migration Reason

Out Migration Request Date
